# Therapeutics for fulminant hepatitis caused by enteroviruses in neonates

**DOI:** 10.3389/fphar.2022.1014823

**Published:** 2022-10-20

**Authors:** Li-Chiu Wang, Huey-Pin Tsai, Shun-Hua Chen, Shih-Min Wang

**Affiliations:** ^1^ School of Medicine, I-Shou University, Kaohsiung, Taiwan; ^2^ Department of Pathology, National Cheng Kung University Hospital, College of Medicine, National Cheng Kung University, Tainan, Taiwan; ^3^ Department of Medical Laboratory Science and Biotechnology, College of Medicine, National Cheng Kung University, Tainan, Taiwan; ^4^ Department of Microbiology and Immunology, College of Medicine, National Cheng Kung University, Tainan, Taiwan; ^5^ Center of Infectious Disease and Signaling Research, National Cheng Kung University, Tainan, Taiwan; ^6^ Department of Pediatrics, National Cheng Kung University Hospital, College of Medicine, National Cheng Kung University, Tainan, Taiwan

**Keywords:** hepatitis, coxsackievirus B, echovirus, antiviral, anti-inflammatory, neonate

## Abstract

Neonatal infection with nonpolio enteroviruses (EVs) causes nonspecific febrile illnesses and even life-threatening multiorgan failure. Hepatitis, which often results in hepatic necrosis followed by disseminated intravascular coagulopathy, is one of the most severe and frequent fatal neonatal EV infection complications. Coxsackievirus B (CVB) 1–5 and many echoviruses have been most commonly identified. Neonatal EV infection treatment has usually involved initial supportive care. Studies for CVB and echovirus infection treatments were developed for more than thirty years. Intravenous immunoglobulin and pleconaril therapy was performed in some clinical trials. Additionally, other studies demonstrated antiviral and/or anti-inflammatory pathogenesis mechanisms of neonatal EV hepatitis in *in vitro* or *in vivo* models. These treatments represented promising options for the clinical practice of neonatal EV hepatitis. However, further investigation is needed to elucidate the whole therapeutic potential and safety problems.

## Introduction

Hepatitis is characterized by jaundice, hepatomegaly, elevated transaminases, and hyperbilirubinemia and is frequently associated with thrombocytopenia and coagulopathy. Acute hepatic necrosis and endothelial injury, liver failure, bleeding complications, and renal failure may ensue (Wang et al., 2001). Acute hepatic necrosis is the most common mechanism of acute liver failure (ALF). ALF in children is a potentially devastating condition that occurs in previously healthy children of all ages. ALF is rare in early infancy, and its detection may be difficult in neonates. Often, a viral-like illness preludes the deterioration into jaundice, coagulopathy, encephalopathy, and death (Alonso et al., 2017). ALF has been invariably associated with high mortality, accounting for 80%–100% (Burdelski, 1994).

ALF is primarily caused by viruses, toxins, or idiopathies (Manns, 1991; Gotthardt et al., 2007). ALF has different etiologies in early infancy than in older children, with a greater contribution from metabolic disorders, neonatal hemochromatosis, hematological malignancies, and hypoxia (McClean et al., 2003; Rademacher et al., 2011). Viral infection accounts for 20%–30% of neonatal ALF (Taylor et al., 2016). Even though hepatitis A, B, and E viruses are the primary cause of ALF, especially in developing countries (Pandit et al., 2015; Manka et al., 2016), infections in neonates are usually asymptomatic and self-limited. Notably, neonates born to HBV carrier mothers with anti-HBe antibodies are prone to develop ALF (Beath et al., 1992). A national immunization program was implemented in 1984 to tackle hyperendemic hepatitis B in Taiwan (Chen et al., 1978). The hepatitis B carrier rate in children covered by the program decreased by 85%, from approximately 15% to <1%, 20 years after the program implementation (Wait and Chen 2012). The success of the hepatitis B vaccine, the most effective method for combating hepatitis B, continues worldwide. Partly, universal hepatitis B vaccination has been justified as ALF prevention in children.

Neonatal ALF may be caused by agents that typically produce mild illness in adults. Herpesviruses, especially herpes simplex viruses, are identified in 50%–70% of neonates with virus-induced ALF (Durand et al., 2001; Verma et al., 2006; Sundaram et al., 2011; Borovsky et al., 2021). Effective anti-HSV drugs are available. However, skin lesions are absent in 30% of neonates with HSV-induced hepatitis, which leads to difficult and often delayed diagnosis. Early diagnosis and prompt anti-HSV therapy are essential to reduce high mortality and morbidity (Verma et al., 2006).

Enterovirus (EV) infections are the most frequently recognized significant pathogens of neonate ALF without available antiviral drugs or vaccines. They account for 3%–50% of virus-induced ALF in neonates (Shanmugam et al., 2011; Sundaram et al., 2011; Bersani et al., 2020; Borovsky et al., 2021). Neonatal infection with EV is not rare. Asymptomatic, recent respiratory or gastrointestinal illness in the mother or family members should include EV infections. Neonates can be infected during the prenatal, intrapartum, or postnatal periods. A sentinel case is thought to occur due to a vertical transmission followed by horizontal transmission to other neonates. Most neonates (79%) who contract EV are typically asymptomatic or have mild illnesses (Jenista et al., 1984). However, 21% of infected neonates develop more severe illnesses, including hepatitis, meningoencephalitis, myocarditis, or fulminant sepsis-like illnesses, and require hospitalization (Jenista et al., 1984; Vergnano et al., 2015). Zhang et al. (2021) reviewed the studies focusing on neonatal EV infections and reported 237 severe cases previously. They demonstrated that 65% of those febrile cases developed hepatitis or coagulopathy. Additionally, 46% of the severe EV cases developed hepatitis or coagulopathy, with a death rate of 27%. Coxsackievirus B (CVB) and echoviruses are identified in more than 80% of EV-infected neonates with fever and hepatitis (Chuang et al., 2019; Zhang et al., 2021).

## Echovirus

The echoviruses, which have been associated with neonatal ALF, lie within the EV taxonomy, especially the echovirus 11 outbreaks (Wang et al., 2001). Since May 2018, the Taiwan Centers for Disease Control was notified of increased reports related to severe EV infection in newborns. Through July 31, severe echovirus 11 infections were confirmed in eight neonatal patients (five males, age 0–12 days). Hepatitis with coagulopathy (*n* = 8), sepsis syndrome (*n* = 8), myocarditis (*n* = 2), and multiorgan dysfunction (*n* = 7) were included as clinical presentations. The mortality was 87.5% (*n* = 7) (Su et al., 2018).

Echovirus can cause disseminated infection with fulminant hepatic failure in neonates (Wang et al., 2001). Severe neonatal disseminated infections with fulminant liver disease have been linked to echovirus types 6, 7, 9, 10, 11, 12, 14, 19, and 30 (Hughes et al., 1972; Mostoufizadeh et al., 1983; Speer et al., 1984; Ho-Yen et al., 1989; Verboon-Maciolek et al., 1997; Pino-Ramírez et al., 2008). Associated virulence factors with certain echovirus serotypes appear as important neonatal infection determinants. Serotype 11 caused 70% of all cases (Modlin, 1986), while echovirus 11 is one of the most isolated serotypes obtained from patients in clinical laboratories. Khetsuriani et al. (2006) reported echovirus 11 (14%) as the leading serotype of commonly isolated EV from neonates from 1983 to 2003 in the United States of America. In Taiwan, the first reported fatal case of echovirus 11 infection was a newborn with fulminant hepatitis and disseminated intravascular coagulopathy (DIC) (Hsiao et al., 2003). Diffuse extensive centrilobular hemorrhagic necrosis and other findings suggestive of DIC are the dominant pathologic findings of echovirus 11 infection (Mostoufizadeh et al., 1983). Scattered foci of dystrophic myocardial calcification, hepatic architecture distortion with fibrous connective tissue surrounding regenerative nodules and large foci of dystrophic calcification, and adrenal hemorrhagic necrosis and calcification are other disseminated findings (Wells et al., 2021).

Wells et al. (2021) reported that human homolog of the neonatal Fc receptor (hFcRn) expression and type I interferon (IFN) signaling ablation are key host determinants that are involved in echovirus pathogenesis, similar to severe hepatitis observed in humans (Bose et al., 1983). Extensive zone 3 hemorrhagic necrosis, endothelial injury, and minimal inflammation were observed in the acute stage, followed by veno-occlusive changes as pathologically evidenced (Ventura et al., 2001).

## Coxsackievirus B

**FIGURE 1:**
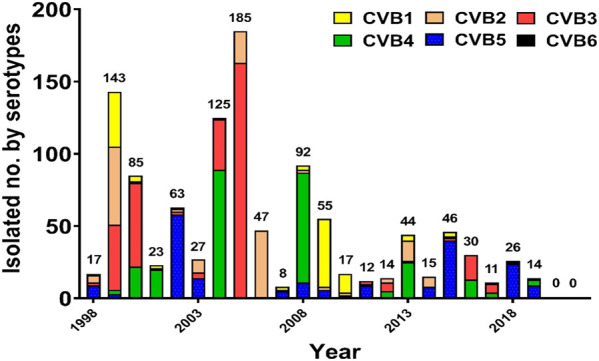
Annual distribution of coxsackievirus B isolates according to the serotype in National Cheng Kung University Hospital, 1998–2021.

Severe neonatal CVB diseases usually occurred in the first 2 weeks of life. A severe CVB neonatal infectious spectrum may be diagnosed as meningitis, encephalitis, meningoencephalitis, myocarditis, hepatitis, pancreatitis, coagulopathy, and pneumonitis (Khetsuriani et al., 2006; Adams et al., 2013). Infants with hepatitis clinically presented with jaundice, hepatomegaly, abdominal distension, and coagulopathy. Laboratory abnormalities included elevated transaminases, hyperbilirubinemia, thrombocytopenia, and evidence of DIC (Wang et al., 1998). Figure 1 for a start shows the serotype distribution of CVB isolates from 1998–2021 in the virological laboratory of the National Cheng Kung University Hospital.

Serum aspartate aminotransferase (AST) and alanine aminotransferase (ALT) are released from necrotic hepatocytes into circulation, causing elevated serum concentrations. Serum AST and ALT concentrations may dramatically increase with acute liver damage progression and subsequently decrease to the normal range by depleting their content in the liver. Soudée et al. (2014) revealed that the risk factors for severe neonatal EV infections included hepatitis (73%), thrombocytopenia (82%), and coagulopathy (64%). Lv et al. (2016) found 18 (13.7%) patients with low platelet counts. The risk of EV infections increases 3.647-fold once the platelet count is < 150 × 10^9^/L. Thrombocytopenia is indicated as a major risk factor for development of severe EV infection in neonates. Bryant et al. (2004) reported two of 10 cases with hepatitis, both of whom had severe hepatitis, thrombocytopenia, and DIC. They had significantly increased liver enzymes and coagulopathy and the association between these features and mortality. Patients had DIC with elevated prothrombin time, activated partial thromboplastin time (APTT), D-dimer, and fibrinogen levels, similar to our previous findings (Bryant et al., 2004). CVB1-5 had the risk of the fatal outcome of EV neonatal hepatitis during the past 30 years in our hospital. However, only CVB4, with a very high (40%) reported case fatality to the National Enterovirus Surveillance System, had a significantly increased risk of death compared with other EV serotypes (Khetsuriani et al., 2006).

Wang et al. (1998) documented fatal neonatal cases of CVB infection and presented clinical and histologic evidence to demonstrate liver involvement and intravascular coagulation. Accordingly, ALF in early infancy has been invariably associated with high mortality. The emerging concept of “immune dysregulation” that leads to aberrant natural killer cell function and cytokine-related liver necrosis has assumed a prominent place in theories related to indeterminate pediatric ALF (Taylor et al., 2016). They further showed reduced blood AST, ALT, and lactate dehydrogenase (LDH) levels and mortality of CVB3-infected mice treated with the coxsackievirus-adenovirus receptor (CAR) antibody (Liu et al., 2013).

## Treatment

Neonatal EV infection treatment focuses on managing the most severe physiological derangements. The mainstay of treatment for severe disease is supportive care. Intravenous immunoglobulin (IVIG) has been therapeutically used against EVs in the clinical settings of neonates (Rotbart, 2000). Previously, Abzug et al. (1995) performed a randomized trial using IVIG and supportive therapy in neonates with EV disease and revealed that IVIG products for the patient’s viral isolate provided a more rapid reduction in viremia than in patients who did not receive IVIG. High antibody concentrations of IVIG to neutralize the specific EV and reduce inflammations may alter the disease course in the infected neonates.

Antiviral therapy for neonatal EV infections is not commercially available. High-throughput screening of natural compounds or FDA-approved drugs provides mounting antiviral candidates. We selectively reviewed those compounds evaluated *in vivo*. Several compounds have been developed that selectively inhibit the interaction of CVB with CAR. The WIN compounds are antiviral drugs that interact with the hydrophobic pocket at the bottom of the canyon. Pleconaril, vapendavir, disoxaril, and pirodavir are the new generation of metabolically stable capsid function inhibitors (Kearns et al., 1999). Among them, pleconaril is the first to exert broad-spectrum anti-EV activity. Pleconaril is considered well-tolerated in treated patients (Abzug et al., 2003; Hayden et al., 2003; Desmond et al., 2006). The compassionate use of pleconaril in patients with life-threatening EV meningitis or meningoencephalitis shows favorable outcomes (Rotbart et al., 2001). However, the randomized, double-blinded, placebo-controlled studies show inconsistent efficacy in treating patients with EV meningitis or meningoencephalitis (Abzug et al., 2003; Desmond et al., 2006). Also, pleconaril might induce adverse effects due to its ability to increase cytochrome P-450 3A (CYP3A) activity (Ma et al., 2006) and reduce plasma levels of CYP3A substrates, including some contraceptive steroids (Hayden et al., 2003). These, taken together, lead to the halted development of pleconaril in clinical use. The NIH-sponsored collaborative antiviral study group performed a randomized, double-blind, placebo-controlled study of the virologic and clinical efficacy, pharmacokinetics, and safety of pleconaril for treating severe neonatal EV sepsis (hepatitis with coagulopathy and myocarditis) and revealed an increased survival among EV-infected persons who received pleconaril due to its potential efficacy for neonatal EV infections (Abzug et al., 2016). Further studies are needed to evaluate the efficacy of pleconaril in treating EV-induced ALF in neonates.

Drugs that interfere with viral and cellular proteins during replication were developed. EV 2C is the most conserved nonstructural protein among EVs and contains RNA helicase activity that is critical for the viral life cycle. Fang et al. (2021) demonstrated that targeting the helicase activity of 2C with a rationally designed peptide is an efficient antiviral strategy, stability, and bioavailability against CVB3 and E11 in the ICR mouse model. They suggested a promising clinical potential in these peptides to be further developed as broad-spectrum antiviral drugs. EV replicates within replication organelles composed of phosphatidylinositol-4-phosphate (PI4P)-enriched membranes from Golgi apparatuses and the endoplasmic reticulum (Hsu et al., 2010; Greninger et al., 2012; Sasaki et al., 2012). The phosphatidylinositol 4-kinase IIIβ (PI4KIIIβ), the kinase responsible for PI4P generation, is a potent broad-spectrum anti-EV target. Oxoglaucine, blocking PI4KIIIβ activity (Arita et al., 2015), and enviroxime, disrupting 3A interaction with PI4KIIIβ, are developed to suppress PI4KIIIβ function. Among them, enviroxime is the first one tested in trials, which, however, is halted at phase II due to the lack of therapeutic benefits in treating rhinovirus-infected patients (Phillpotts et al., 1983; Miller et al., 1985).

Repurposing existing medications would facilitate the development of antiviral therapy for EV. Licensed antiviral agents for treating highly pathogenic RNA virus infections included anti-influenza medications, M2 channel inhibitors (amantadine and rimantadine), and neuraminidase inhibitors (oseltamivir). Ribavirin is licensed for treating respiratory syncytial virus and hepatitis C virus infections (De Clercq, 2004). Park et al. (2011) revealed that amantadine and ribavirin exhibited antiviral activity against the Korean E18 strain, with amantadine showing stronger effects than ribavirin.

The high mutation rate (10^–3^ per nucleotide) of EV leads to the inevitable development of drug resistance and limits the general use of antiviral drugs in patients. For instance, in the trial using pleconaril to treat common colds, rhinoviruses with reduced susceptibility or complete resistance to pleconaril were isolated in 10.7% and 2.7% of drug-treated patients, respectively (Pevear et al., 2005). A single mutation in the viral 2C protein is sufficient to confer resistance against the antiviral treatment but was only investigated in *in vitro* experiments (De Palma et al., 2008). Combining two or more drugs with synergistic effects or distinct antiviral mechanisms is proposed to overcome the issue. Serial studies show that consecutive alternating administration of a WIN-like compound, pleconaril or disoxaril, followed by two replication inhibitors, guanidine hydrochloride or MDL-860 and oxoglaucine, protects mice from lethal CBV infections (Stoyanova et al., 2015a; Stoyanova et al., 2015b; Vassileva-Pencheva et al., 2016). Notably, pleconaril increases the cytochrome P-450 3A (CYP3A) enzyme activity (Ma et al., 2006) and might reduce other drug efficacy in combined use. Whether drug efficacy is diminished in combined use with pleconaril should be evaluated. Stoyanova et al. (2015a) showed that the triple combination (pleconaril, guanidine-HCl, and oxoglaucine) increases the barriers to drug resistance without altering drug sensibility. These studies demonstrate a promising strategy to treat fatal EV infection, which requires further investigation.

Another strategy to overcome drug resistance is targeting cellular proteins, especially those with pro-viral or immune-modulatory functions, since cellular targets are unlikely to mutate in response to therapies. Our previous studies of the liver in CVB-infected mice histopathologically demonstrated polymorphonuclear cell infiltration, massive hepatocyte necrosis, and apoptosis (Liu et al., 2013). Wang et al. (2019) showed that CVB infection decreased programmed cell death protein 1 (PD-1) ligand expression. Furthermore, systemic PD-L1 (B7H1; CD274) and PD-L2 (B7-DC; CD273) treatment markedly augmented the apoptosis of proliferating lymphocytes and alleviated injury in the cardiomyocytes caused by CVB infection. They suggested the PD-1 pathway as a potential immunotherapeutic target for CVB infection prevention and treatment.

Natural compounds, a large majority coming from plant origin, have enormously contributed as immunomodulatory therapeutics. Natural medicines have always fascinated humans with their quality of harmless treatments with minimal side effects. There are thousands of natural compounds, such as the compounds and their derivatives, which are known to influence the immune system either by affecting the immune cell functions, such as dendritic cells, macrophages, lymphocytes, and natural killer cells, or by affecting the antibody secretion to control the infection and maintain immune homeostasis (Bragg et al., 2018). Oroxylin A is an O-methylated flavone that is found in the medicinal plant *Scutellaria baicalensis* (Tebruegge et al., 2009). Several previous studies have shown broad-spectrum antiviral activities of extracts and compounds from *S. baicalensis*. Oroxylin A treatment mitigated the histological lesions and apoptotic cell death, reduced viral titers, and decreased the serum levels of interleukin-6 and tumor necrosis factor-α in CVB3-infected mice (Kwon et al., 2016). *Saururus chinensis* (Lour.) Baill is a folk medicine that had antiviral effects on HIV-1 (Lee et al., 2010), HSV (Tian et al., 2012), and EV71 (Wang et al., 2015). *Schisandra chinensis* Baill extract exerted significant antiviral activity against CVB3 infection in vero cells and demonstrated manassantin B (Man B) as one of the antiviral components. Man B protected from systemic infection of CVB3 and exerted antiviral activity by activation of the STING/TBK-1/IRF3 signaling pathway. Furthermore, Man B reduced CVB3-mediated inflammatory cytokine production in mice (Song et al., 2019). Ethyl 3-hydroxyhexanoate (EHX) is a fatty acid ethyl ester of 3-hydroxyhexanoic acid, which is a key volatile compound in several fruits and is a potent antiviral compound (Medina et al., 2020). Olasunkanmi et al. (2022) demonstrated that EHX treatment significantly inhibits CVB replication both *in vivo* and *in vitro*.

EV epidemics have highlighted the public health impact in Taiwan. The recurrences of known serotypes and the evolution of additional new variants may be anticipated. CVBs and echoviruses are one of the most important infectious agents associated with acute hepatitis in neonates. Both viral and immune mechanisms are involved based on further understanding of the hepatic injury pathogenesis caused by CVBs and echoviruses. Antiviral, anti-inflammatory therapy and the potential combination with immunomodulatory therapies may hold the greatest benefits for improving the outcome of CVBs and echovirus hepatitis. An extensive future investigation is required concerning their mechanisms of action at the systemic, cellular, and molecular levels to extend up to broad-spectrum clinical trials.
